# DNA Polymorphisms of the Lipoprotein Lipase Gene and Their Association with Coronary Artery Disease in the Saudi Population

**DOI:** 10.3390/ijms13067559

**Published:** 2012-06-18

**Authors:** Abdulaziz A. Al-Jafari, Mohamed S. Daoud, Abdulelah F. Mobeirek, Mohammad S. Al Anazi

**Affiliations:** 1 Department of Biochemistry, Faculty of Science, King Saud University, 11451, Saudi Arabia; E-Mails: dr_mohamed301@hotmail.com (M.S.D.); msanazi@ksu.edu.sa (M.S.A.A.); 2 Cardiac Sciences Department, Faculty of Medicine, King Saud University, 11472, Saudi Arabia; E-Mail: amobeirek@yahoo.com

**Keywords:** coronary artery disease, lipoprotein lipase, genotypes, restrictions fragment, Saudi populations and polymorphism

## Abstract

Coronary heart disease (CHD) is a major health problem and a major cause of death in most countries. Evidence has been presented that gene polymorphisms (*Hin*dIII, *Pvu*II and Ser447Ter) of lipoprotein lipase (LPL) are risk factors of coronary artery disease (CAD).

**Aim:**

Our objective of the present investigation was to determine whether 3 LPL polymorphisms (LPL-*Hin*dIII, LPL-*Pvu*II and LPL-Ser447Ter) can be considered as independent risk factors for CAD in the Saudi population.

**Methods:**

We recruited 120 CAD subjects, confirmed angiographically with identical ethnic backgrounds and 65 control subjects. Polymerase chain reaction-restriction fragment length polymorphisms (RFLP) technique was used to detect the polymorphisms of the LPL gene.

**Results and conclusion:**

For the *Hin*dIII genotype, within the CAD group, the frequencies of the H^+^H^+^ were found in 50.8%, whereas 44.2% carried the H^−^H^+^ genotype, and 5% carried the H^−^H^−^ genotype. Within the control group, the H^+^H^+^ genotype was found in 44.6%, whereas 35.4% carried the H^−^H^+^ genotype, 20% carried the H^−^H^−^ genotype. The odds ratio (OR) of *Hin*dIII genotype H^+^H^+^ *vs.* H^−^H^−^ genotype at 95% Confidence Interval (CI) were 4.6 (1.57–13.2) and *p* < 0.005, hence showing no significant association with CAD. For the *Pvu*II genotype, within the CAD group the frequencies of the P^+^P^+^ found in 41.7% whereas 43.3.2% carried the P^−^P^+^ genotype, and 15% carried the P^−^P^−^ genotype. Within the control group the P^+^P^+^ was found in 38.5%, 43.0% carried the P^−^P^+^ genotype, and 18.5% carried the P^−^P^−^ genotype. The OR of *Pvu*II genotype P^+^P^+^ *vs.* P^−^P^−^ genotypes (95% CI) is 1.33 and *p* = 0.52; hence, it was also insignificant to show association with the disease. For the Ser447Ter genotype, within the CAD group, the frequencies of the C/C found in 83.3%, whereas 16.7% carried the C/G genotype. Within the control group, the C/C was found in 87.7% and 12.3% carried the C/G genotype. We did not get any GG genotypes in control as well as patients for this gene. It can be concluded that C allele of gene masks the presence of G allele in the Saudi population. The OR of CG + GG *vs.* CC (95% CI) is 1.43 from 0.59 to 3.44 which is insignificant. Hence this gene also has no significant association with CAD in the Saudi population.

## 1. Introduction

In recent years, the relationship between the atherosclerotic cardiovascular disease, predominantly coronary artery disease (CAD) and many genetic variants in different populations are very well documented. CAD is one of the greatest causes of morbidity and mortality in all over the world being responsible for a high percentage of death annually in different countries [1,2]. It has been well known for decades that classical risk factors for CAD such as smoking tobacco or disorder in lipid metabolism increase its occurrence [3,4]. CAD shows strong familial aggregation, especially when it occurs at an early age and when many relatives are affected [5]. Lipid disorders have been associated as a well-known risk factors for CAD; however, only a small percentage of CAD patients have recognized monogenetic disease due to dysfunctional mutations in lipoproteins or lipoprotein-related genes (e.g., catabolic enzymes, receptors) [5]. Therefore, various risk factors remain unexplained [5,6] and this is believed to be due to the interaction of multi-factorial inheritance and environmental factors (e.g., diet, exercise, smoking) [5]. In this regard common variants of genes central to lipid metabolism that are associated with modest changes in protein function might be essential contributors to risk at a population level, maybe in association with other specific environmental and genetic factors [5,7]. The lipoprotein lipase (LPL) gene represents an excellent candidate to explain parts of the genetically determined risk of atherosclerosis [8]. Mature LPL is a 448 amino acid glycoprotein with chromosomal location 8p22 [9,10]. It is one of about 14 genetically mapped, lipid-related proteins known to be capable of contributing to the incidence of CAD [5]. Lipoprotein lipase, strategically anchored to vascular endothelium, plays a central role in lipid metabolism. Lipoprotein lipase-catalyzes hydrolysis of triglycerides of exogenous (chylomicron) and endogenous very low density lipoproteins (VLDL) origins to provide free fatty acids for oxidation in the heart and other tissues and for storage in adipose tissue [11]. Lipoprotein lipase is multifunctional, and is documented to have been involved as a ligand for low density lipoprotein (LDL) receptor-related protein and influences the hepatic secretion and uptake of VLDL and LDL cholesterol [12]. It is well known that rare LPL mutations cause marked dyslipidemias (e.g., familial LPL deficiency with chylomicronemia [13], at least some of which are known to be involved in the development of premature atherosclerosis [13]. However, we were interested in exploring the relationship between LPL polymorphisms and the risk for CAD by detecting the frequencies of LPL *Hin*dIII, *Pvu*II and Ser447Ter genotypes in the Saudi population. Several polymorphic variants of the LPL gene have been identified in recent years and evaluated for their effects on plasma lipids and atherosclerosis risk from different populations [14–18]. Those defined by the *Hin*dIII, *Pvu*II and Ser447Ter restrictions fragment length polymorphism sites, located on introns 8, 6 and 9 respectively, of the LPL gene, are common and may be associated with subtle alterations in plasma lipids. Various studies on *Hin*dIII, *Pvu*II and Ser447Ter polymorphisms and their association with diseases related to CAD have been previously reported. The *Hin*dIII, *Pvu*II and Ser447Ter polymorphisms have been variably reported to associate with CAD [14–20]. However, the results are inconsistent [17,18,21]. Therefore, given the importance of LPL as a candidate gene for CAD risk, we have evaluated independent Saudi and well defined, ethnically relatively homogenous and angiographically controlled Saudi population to determine whether the *Hin*dIII, *Pvu*II and Ser447Ter polymorphisms are associated with the risk of the development of CAD.

## 2. Results and Discussion

Characteristics of the 185 study subjects (120 CAD) patients and (65 control subjects) are presented in Table 1. Plasma level of Fasting blood sugar (FBS), triglycerides (TG), total cholesterol (TC) and low density lipoprotein-cholesterol (LDL-c) tended to be higher and HDL-c lower in CAD patients than in controls at admission. Table 1 shows the plasma level of fasting blood glucose, triglycerides, total cholesterol and low density lipoprotein cholesterol were significantly higher in CAD group (*p* < 0.001) as compared to normal control group.

Many studies have focused on the relationship between LPL gene polymorphism and the incidence of cardiovascular disease in various populations [14,16,19,21,22]. Some studies investigated the influence of LPL gene activity on levels of plasma TG, TC, HDL-c and apolipoproteins, but the results are inconsistent [17,22–26]. Clee *et al.* (2000) [23] demonstrated that decreased plasma LPL activity is associated with high TGs and low HDL phenotypes in patient samples and Ser447Ter mutation is associated with higher plasma LPL activity. Given the importance of LPL as a candidate gene for CAD risk, we evaluated a reasonably small, independent, well-defined, angiographically controlled Saudi population to determine whether the LPL-*Hin*dIII, LPL-*Pvu*II and LPL-Ser447Ter polymorphisms were associated with defined CAD in individuals of Saudi origin.

Diabetes mellitus, dyslipidemia, hypertension and smoking, were selected as risk factors. The frequencies of major CAD risk factors are summarized in Table 2. Diabetes mellitus, dyslipidemia, hypertension, and smoking were more frequent in the CAD patient group than in controls. Diabetes mellitus, dyslipidemia and hypertension were found to be risk factors for CAD (Odds 17.8, 6.4, and 18.02 respectively, *p* < 0.0001) whereas no such association was observed for smoking (odds 1.49 *p* = 0.31). Coronary artery disease (CAD) is a multifactorial disorder that is thought to result from an interaction between genetic back ground and environmental factors such as diet, smoking, and physical activity. It is usually associated with conventional risk factors, including hypertension, diabetes mellitus, and hypercholesterolemia [27]. In general, individuals with hypertension, diabetes mellitus, and hypercholesterolemia and those with none of these factors are considered at high and low risk, respectively, for development of CAD. Age, smoking, hypertension, and diabetes have been established as independent risk factors for ischemic CVD [28]. Hypertension and dyslipidemia were found to influence CAD [29]. Genetic factors were statistically independent of age, smoking, or hyperuricemia, as well as hypertension, diabetes mellitus, and hypercholesterolemia. The results indicated that smoking is an important environmental factor for CAD in low-risk men, consistent with the notion that the cessation of smoking is important in the prevention of CAD in these individuals [28].

The polymorphic allele displaying the restriction site were designated with a plus sign (+) and the allele without the site with a minus sign (−). The identified genotypes were named according to the presence or absence of the enzyme restriction sites, *Hin*dIII and *Pvu*II (+/+), (+/−), and (−/−) are homozygote’s for the presence of the site, heterozygote’s for the presence and absence of the site, and homozygote’s for the absence of the site, respectively.

**Figure 1:**
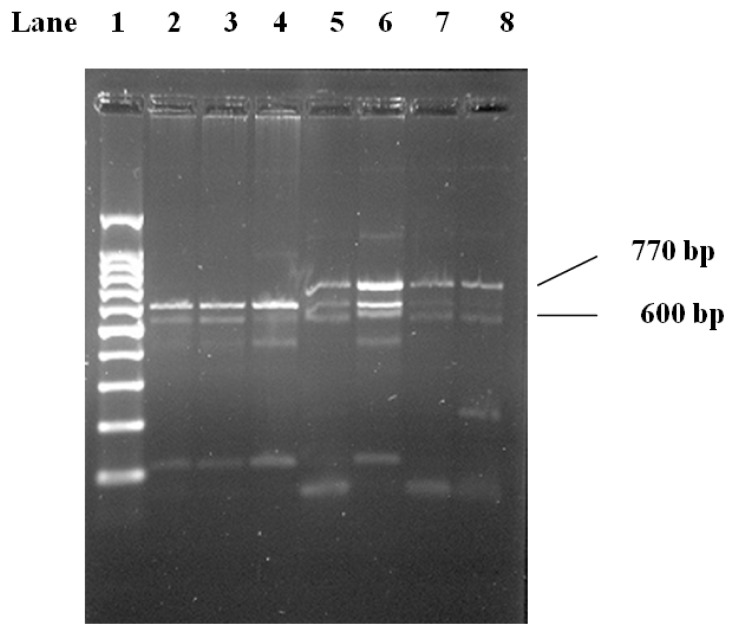
2.5% Agarose gel electrophoresis cutting with *Hin*dIII. Lane 1, 100 bp DNA molecular weight marker. Lanes 2–8, lipoprotein lipase (LPL) PCR product digested with *Hin*dIII. Homozygosity for presence (lanes 2–4), heterozygosity (lanes 5, 6), and homozygosity for absence (lanes 7, 8) of restriction site are shown.

The *Hin*dIII genotypes were determined by PCR and digestion using *Hin*dIII restriction enzyme. When present, the *Hin*dIII resulted in fragments of 600 bp after digestion (Figure 1). The LPL-*Hin*dIII genotypes were determined according to Anderson *et al.* 1999 [1].

**Figure 2:**
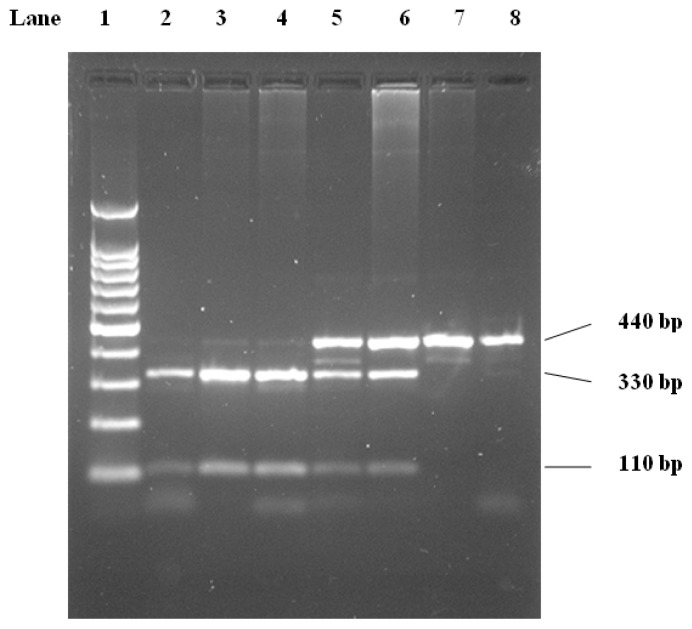
2.5% Agarose gel electrophoresis cutting with *Pvu*II. Lane 1, 100 bp DNA molecular weight marker. Lanes 2–8, LPL PCR product digested with *Pvu*II. Homozygosity for presence (lanes 2–4), heterozygosity (lanes 5, 6), and homozygosity for absence (lane 7, 8) of restriction site are shown.

The *Pvu*II genotypes were determined by PCR and digestion using the *Pvu*II restriction enzyme. When present, *Pvu*II produces fragments of 330 and 110 bp (Figure 2). The LPL-*Pvu*II genotypes were determined according to Anderson *et al.* 1999 [1].

**Figure 3:**
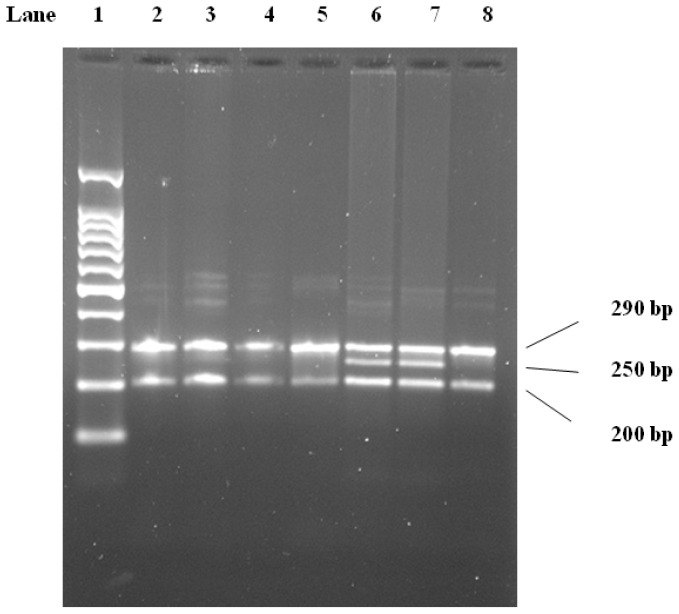
2.5% Agarose gel electrophoresis cutting with *Mnl*I. Lane 1, 100 bp DNA molecular weight marker. Lanes 2–8, LPL PCR product digested with *Mnl*I. Homozygosity for presence lanes 2–5 and 8, heterozygosity lanes 6, 7 of restriction site are shown.

The Ser447-Ter genotypes were determined by PCR and digestion using the *Mnl*I restriction enzyme. The identified genotypes were named to the presence or absence of the enzyme restriction sites, so Ser447-Ter GG, CG, and CC are homozygote for the presence of the site, heterozygote for the presence and absence of the site, and homozygote for the absence of the site, respectively. The PCR product of 448 bp contains 2 *Mnl*I restriction sites, one of which is a polymorphic site indicating the Ser447-Ter mutation. Digestion of the PCR product with *Mnl*I resulted in three fragments of 290, 250, and 200 bp (Figure 3). LPL-Ser447-Ter mutations were determined according to Sawano *et al.* 2001 [18].

### Genotype Distribution and Allele Frequency of the LPL-*Hin*dIII, LPL-*Pvu*II and LPL-Ser447Ter

Genotype frequencies did not deviate from Hardy-Weinberg expectations in both controls and CAD group. The genotype distribution and the allele frequency for LPL gene polymorphisms are summarized in Table 2. H^+^ and H^−^ alleles did not differ significantly between CAD group and controls. For the *Hin*dIII genotype, within the CAD group (*n* = 120), the frequencies of the H^+^H^+^ found in 61 individuals (50.8%), whereas 52 (44.3%) carried the H^−^/H^+^ genotype, and 6 (5%) carried the H^−^/H^−^ genotype. Within the control group (n = 65), the H^+^/H^+^ genotype was found in 29 (44.6%), whereas 23 (35.4%) carried the H^−^/H^+^ genotype, and 13.0 (20%) carried the H^−^H^−^ genotype. the distribution of *Hin*dIII demonstrated that CAD patients had lower H^−^H^−^ frequency compared to the control group and higher H^+^H^−^, H^+^H^+^ frequencies compared to the control group Table 3. Odds Ratios were 4.5 and 4.6 respectively when compared with (H^−^H^−^) genotype. Individuals with homozygous (H^+^H^+^) or (H^+^H^−^) genotype were at much higher risk of developing CAD compared to the (H^−^H^−^) genotype (*p* < 0.005), Table 4. For the *Pvu*II genotype, within the CAD group (*n* = 120), the frequencies of the P^+^P^+^ found in 50 (41.7%), whereas 52 (43.2%) carried the P^−^P^+^ genotype, and 18 (15%) carried the P^−^P^−^ genotype. Within the control group (*n* = 65), the P^+^P^+^ was found in 25 (38.5%), 28 (43.0%) carried the P^−^P^+^ genotype, and 12.0 (18.5%) carried the P^−^P^−^ genotype. The distribution of *Pvu*II polymorphism demonstrated that CAD patients had higher P^+^P^−^, P^−^P^−^, P^+^P^+^ frequencies compared with controls, Table 3. The odds ratios of *Pvu*II genotype P^−^P^−^ *vs.* P^−^P^+^ and P^+^P^+^ genotypes (95% CI) is 1.26 (90.53–2.99) and 1.33 (0.56–3.20) respectively, hence showing no significant association with CAD disease Table 5. In previous studies, the prevalence rate of the LPL-*Hin*dIII (H^+^H^+^) genotype was implicated as the most common genotype associated with CAD. Shimo-Nakanishi *et al.* (2001) [17] demonstrated that *Hin*dIII polymorphism is associated with increased risk of atherothrombotic infarction. Likewise, Chamberlain *et al*. (1989) [30] indicated that there were differences in frequencies of both *Hin*dIII and *Pvu*II alleles between Whites and Japanese populations, the statistical analysis for the LPL-*Hin*dIII genotypes indicates significant differences in the distribution of these genotypes between the control groups and the CAD groups, which in agreement with previous reported study [20]. Several studies have investigated the possibility that the LPL*-Hin*dIII and the LPL-*Pvu*II polymorphisms are associated with the severity of CAD in various populations [1,14,16,19,20,31–36]. However, limited data are available concerning the Saudi population [21], which has not adequately defined the association between LPL polymorphism and CAD. While a large number of studies have found the LPL-*Hin*dIII (H^+^H^+^) genotype to be associated with CAD, the finding from the association studies with LPL-*Pvu*II polymorphisms have been inconsistent [14,15,19,33,35]. For example, Thorne *et al.* (1990) [15] reported an increase in the LPL-*Hin*dIII (H^+^H^+^) genotypic frequencies in patients with coronary atherosclerosis in Japanese population whereas Peacok *et al.* (1992) [33] observed no differences in either LPL-*Hin*dIII or LPL-*Pvu*II allelic frequencies in young survivors of myocardial infarction in Spanish population. Ukkola *et al.* (1995) [35] found reductions in various measures of ischemic heart disease in non-insulin-dependent diabetics who were LPL-*Pvu*II (P^+^P^+^), but noted increases in those who were LPL-*Hin*dIII (H^−^H^−^) in Canadian adult population. In contrast, Gerdes *et al.* (1995) [34] found the LPL- *Hin*dIII (H^+^H^+^) genotype to be positively associated with a family history of premature ischemic heart disease in a group of Danish men. Similarly, Mattu *et al.* (1994) [32] found an association between the LPL-*Hin*dIII H^+^H^+^ genotype and CAD, but not between the LPL-*Pvu*II (P^+^P^+^) genotype and CAD in native Brazilian population. On the other hand, Wang *et al.* (1996) [14] reported an association between the extent, but not occurrence of CAD and the LPL-*Pvu*II (P^+^P^+^) genotype. However, in the same study, Wang *et al.* (1996) [14] found no association between the LPL-*Hin*dIII genotype and CAD. Interestingly, Gambino *et al.* (1999) [31] found a striking association between the LPL-*Hin*dIII (H^+^H^+^) genotype and multi-vessel disease in an Italian group of young survivors of myocardial infarction. Also it has been reported that Anderson *et al.* (1999) [1] found a moderate association between the LPL-*Hin*dIII (H^+^H^+^) genotype and CAD, whereas the LPL*-Pvu*II (P^−^P^−^) genotype was modestly associated with CAD. In summary, the relevance of these genotypes may vary among different populations.

For the Ser447Ter genotype, within the CAD group (*n* = 120), the frequencies of the CC found in 100 (83.3%), whereas 20 (16.7%) carried the CG genotype. Within the control group (*n* = 65), the CC was found in 57 (87.7%) and 8 (12.3%) carried the CG genotype. We did not get any GG genotypes in the control or any patients for this gene. The homozygote of G allele was incorporated with the heterozygote due to the low frequency of GG, higher frequencies of CC and CG genotypes in the CAD group Table 3. OR of CC *vs.* CG+GG (95% CI) is 1.43 from 0.59 to 3.44, which is insignificant, Table 4. Interestingly, no GG genotype was observed in the CAD and control groups in the distribution of Ser447Ter polymorphism in the present study. However, we found for the first time in Saudi populations new genotype frequencies of Ser447-Ter mutations of the LPL gene between the CAD patients and the healthy controls. In previous reported studies, the frequency of the Ser447-Ter genotype of GG and CG was significantly lower in a group of Japanese CAD patients than in the control [37], representing a significantly low risk of CAD. Also Shimo-Nakanishi *et al.* (2001) [17] found that Ser447 Stop mutations of the LPL gene are a novel genetic marker for low risk of atherothrombotic cerebral infarction. Kozaki *et al*. (1993) [37] originally found an approximately 2-fold increase in the enzymatic activity of LPL resulting from the Ser447-Ter mutations at the carboxy terminal of LPL transfected and expressed *in vitro*, conflicting results that demonstrated that Ser447-Ter mutations might have either no or an inverse effect on LPL function may have been caused by different methodology.

Haplotype frequencies for the *Hin*dIII, *Pvu*II and Ser447Ter alleles were estimated for controls and patients (Table 6). A score for each haplotype (Hap-score) was calculated and the *P*-value was obtained for the significance of each Hap-score. The haplotypes −/−/C, +/−/C, −/−/G, −/+/C, −/+/G, +/−/G, +/+/C and +/+/G are the most common in the CAD patients group. There were differences between the CAD group and control group with regards to the haplotype distribution. Comparison of the common haplotype frequencies between the CAD patients group and control group revealed a significant increase in the frequency of the most common haplotype in those with CAD. This also suggests an increase in the frequency of less common haplotypes among some cases. Haplotypes −/−/G and +/+/C were associated with a significantly increased risk factor of CAD (*p* = 0.06, 0.04 respectively). Table 6 Shows that combined genotypes may play an important role in the process of atherogenesis. It has been proposed that the H^−^allele is a marker either for a mutation that alters an amino acid within LPL, causing enzyme activity or lipid binding to be more efficient, or possibly for a sequence change within the promoter, causing higher levels of LPL expression [20]. The frequencies of H^−^/C and P^−^/C were significantly higher in CI patients than in controls, and the H^−^/P^−^/C combined genotype was the most common genotype of *Hin*dIII/*Pvu*II/Ser447Ter combined genotypes in the cerebral infarction group. *P*^−^allele is more frequent and G allele is less frequent in cerebral infarction (CI) patients than in controls. The data also provide strong evidence of an association between polymorphisms in the LPL gene with lipid levels. Moreover, H^−^/C and P^−^/C mutations, especially H^−^/P^−^/C mutation of the LPL gene, are relevant to CI. *Pvu*II polymorphism is a risk factor for CI while Ser447Ter mutation is a protective factor, indicating that the LPL polymorphism remains a useful genetic marker for predicting CI risk in the Chinese [19]. Goodarzi *et al.* 2003 [38] showed no differences between cases and controls, in comparison of genotype frequencies except for a modestly significant difference for the 8393 (*Hin*dIII) variant (*p* = 0.05). However, comparison of the common haplotype frequencies between the Mexican-Americans with and without coronary artery disease revealed a significant decrease in the frequency of the most common haplotype in those with disease. Goodarzi *et al.* 2003 found that six markers in the 3′ end of *LPL* allowed us to distinguish the most common haplotypes occurring in two major U.S. ethnic groups, Hispanic and non-Hispanic Caucasians. The allele and haplotype frequencies were different between the Mexican-Americans and non-Hispanic Caucasians. Furthermore, within the Mexican-Americans, there were positive and negative associations of particular haplotypes with coronary artery disease, suggesting a role of this gene in contributing to CAD in this population. In comparing two different ethnic groups, Goodarzi *et al.* 2003 [38] found several differences in the allele and haplotype frequencies observed in the 3′*LPL* markers. Such differences may affect the results of association studies conducted in different populations.

## 3. Experimental Section

### 3.1. Patient and Control Population

Study patients and control subjects consisted of 120 patients (58 male and 62 female, aged 36–80 years) who were admitted to Department of Cardiology, King Khalid University Hospital, Riyadh, Saudi Arabia, and 65 healthy subjects (29 male and 36 female, aged 32–80) who had no history of CAD as control. Included subjects were of unrestricted age and gender who gave written informed consent to draw blood at the time of angiography or at the time of screening for research deoxyribonucleic acid (DNA) extraction to be used in studies approved by the hospital’s institutional review board and was conducted in accordance with the guidelines set by the ethics committee of College of Medicine and Research Centre (CMRC) of King Saud University, Riyadh, Saudi Arabia. All subjects enrolled in this study were Saudi residents with similar dietary pattern. Key demographic data of subjects were recorded including age and gender lipid profile. Assessment of CAD was made by review of angiograms by the patient’s cardiologist.

### 3.2. Ethical Approval

This study was conducted after review and approval of the Institutional Review Board of the Ethics Committee at KKUH (King Khalid University Hospital), and all subjects gave written informed consent prior to participation.

### 3.3. Plasma Lipid Measurements

Blood samples for the glucose and lipid measurements were withdrawn from the patients and the control subjects after an overnight fast. The plasma glucose concentration was measured by the glucose oxidase method using a Biotrol Kit on a Bayer opera analyzer. Serum total cholesterol was measured using the Biotrol commercial Kit, HDL cholesterol was determined with a commercial Randox Kit, LDL cholesterol was calculated by the formula of Friedwald, and triglyceride determination was made by the method of Lipase/Glycerol Kinase UV endpoint on the opera analyzer.

### 3.4. DNA Extraction

Genomic DNA was extracted from peripheral blood specimens, which were in tubes containing EDTA, which performed as an anticoagulant, using QIAamp DNA isolation Kit from QIAGEN (Germany).

### 3.5. Genotyping

Genotyping was performed by polymerase chain reaction (PCR). The primer sets were selected on the basis of previously published information (18): *Hin*dIII, forward primer (H1), 5′-TGA AGC TCA AAT GGA AGA GT-3′, reverse primer (H2): 5′-TAC AAG CAA ATG ACT AAA-3′; *Pvu*II, forward primer (P1): 5′-ATG GCA CCC ATG TGT AAG GTG-3′; reverse primer (P2): 5′-GTG AAC TTC TGA TAA CAA TCT C-3′; Ser447Ter, forward primer: 5′-TAC ACT AGC AAT GTC TAG GTG A-3′, reverse primer: 5′-TCA GCT TTA GCC CAG AAT GC-3′. Genomic DNA template 3 μL (150 ng) was added to PCR reaction mixture composed of 12.5 μL 2× Promega master mix, 2 μL each primer and distilled water to a final volume 25 μL. The PCR condition were as follows: initial denaturation at 94 °C for 2 min were followed by 40 cycles of denaturation at 94 °C for 15 s, annealing at 50 °C for 30 s, and extension at 72 °C for 1 min, with final extension at 72 °C for 2 min in My Cycler (Bio-Rad).

### 3.6. *LPL* Gene Polymorphism Analysis

Digestion of PCR products was performed by addition of 1 μL of the respective restriction enzyme (*Hin*dIII: Promega, Madison WI, USA; *Pvu*II: Promega, Madison WI, USA, *Mnl*I: England Biolabs, Inc.) to 10 μL of PCR products in 2 μL 10× buffer solution (final reaction volume = 20 μL), and centrifuged for 2 min at the speed of 5000 rounds/min, and aqueous bathed at 37 °C for 4 h. The resulting fragments were resolved by electrophoresis (80 V, 60 min) on 2.5% agrose gel, and visualized on UV light.

The *Hin*dIII site (intron 8) produces a 600-bp fragment after digestion (Figure 1). The *Pvu*II restriction site (intron 6) yields 330-bp and 110-bp fragments (Figure 2). Genotypes were scored by an experienced reader blinded to clinical and angiographic results. Our convention was to refer to the polymorphic allele displaying the restriction site as (+) and the allele without the site as (−). For both *Pvu*II and *Hin*dIII, the (−) allele was less common R [1].

The PCR product of 488 bp contains 2 *Mnl*I restriction sites, of which one is a polymorphic site indicating the Ser447Ter mutation. Digestion of the PCR product with *Mnl*I resulted in 3 fragments of 290, 250, and 200 bp. [17,18]. The identified genotypes were named according to the presence or absence of the enzyme restriction sites, so Ser447Ter GG, CG, and CC are homozygote for the presence of the site, heterozygote for the presence and absence of the site, and homozygote for the absence of the site, respectively (Figure 3).

### 3.7. Statistical Analysis

Measurement data were summarized by mean ± standard deviation (S.D.), and compared with two-sample *t*-test. Enumeration count data were summarized as number (%) and compared with chi-square test (*χ*^2^ test). To analysis was used to evaluate the allelic and genotypic frequencies that were calculated from the observed genotypic counts and to assess the Hardy-Weinberg equilibrium expectations. The same methodology was applied to comparisons between allelic and genotypic frequencies. Associations were determined as odds ratios (ORs) and 95% confidence intervals (CIs). (The odds of carrying a specific allele are defined as the frequency of subjects in whom it occurs divided by the frequency of subjects in whom it does not occur. The odds ratio for the LPL genotype distribution *χ*^2^ analysis was performed. A CAD is the odds of allelic carriage in the diseased [CAD] group divided by the odds in the undiseased [control] group.) Univariate logistic regression was used to determine adjusted ORs for the genetic markers, conditioned on the major CAD risk factors. (*p* value < 0.05 was considered statistically significant). Statistical analyses were performed with the Statistical Package for Social Sciences for Windows, version 11.5 (SPSS, Inc, Chicago, IL, USA).

## 4. Conclusions

We found association of *Hin*dIII polymorphism with CAD, and no association of *Pvu*II and Ser447Ter polymorphisms with CAD. There were no significant differences in the serum levels of TC, TG, HDL-c and LDL-c with H^−^H^−^, H^−^H^+^, H^+^H^+^ genotypes (Table 4). Also P^−^P^−^, P^−^P^+^, P^+^P^+^ and CC and CG+GG alleles did not correlate significantly with plasma levels of TC, TG, HDL-c and HDL-c hence showing no association between these polymorphisms and CAD. Furthermore, our study did not show any protective role of H^+^ P^+^ or G allele (Table 3) against the onset of the disease. Hence, CAD may be a complex disorder of combination of both genetic and environmental factors that may influence the onset of disease and certainly needs more investigation.

## Figures and Tables

**Table 1:** Characteristic of Controls and coronary artery disease (CAD) Patients.

Characteristic	Controls *n* = 65	CAD group *n* = 120	*p*-value
Age, year			
Mean ± SD	49.02 ± 19.73	61.94 ± 10.78	<0.001
Range	(17.0–78.0)	(31.0–89.0)	
Gender			
Male, %	29(44.6)	58(48.33)	<0.001
Female, %	36(55.4)	62(51.67)	
FBS, mmol/L			
Mean ± SD	4.54 ± 0.58	7.79 ± 3.68	<0.001
Range	(3.26–5.62)	(3.3–20.6)	
TG, mmol/L			
Mean ± SD	1.06 ± 0.28	1.56 ± 0.70	<0.001
Range	(0.53–1.72)	(0.57–3.77)	
TC, mmol/L			
Mean ± SD	3.80 ± 0.48	4.24 ± 1.09	<0.001
Range	(3.01–5.11)	(0.77–7.5)	
HDL-c, mmol/L			
Mean ± SD	1.26 ± 0.39	1.11 ± 0.88	N.S
Range	(0.76–2.11)	(0.53–5.1)	
LDL-c, mmol/L			
Mean ± SD	1.65 ± 0.33	2.53 ± 0.91	<0.001
Range	(1.0–2.5)	(1.01–4.89)	

Data are represented as mean ± SDs for all quantitative traits. Student’s *t*-test and *χ*^2^ test were used to compare the values of controls and CAD patients.

**Table 2:** Risk Factors for Coronary Artery Disease in Patients and Control Subjects.

Paramerter	CAD *n* = 120	Control *n* = 65	OR	95% CI	*p* *
Diabetes mellitus					
Diabetic	82(68%)	7(11%)			
Non diabetic	38(32%)	58(89%)	17.8	7.46–42.8	0.0001
Dyslipidemia					
Positive	65(54%)	12(18%)			
Negative	55(46%)	65(82%)	6.4	3.14–13.05	0.0001
Hypertension					
Hypertensive	86(72%)	8(12%)			
Normotensive	34(28%)	57(88%)	18.02	7.78–41.73	0.0001
Smoking					
Smoker	28(23%)	11(17%)			
Nonsmoker	92(77%)	54(83%)	1.49	0.69–3.24	0.31

* *p* < 0.05;

CI = confidence interval.

**Table 3:** Genotype distributions and allele frequency of the *Hind*III, *Pvu*II and Ser447Ter.

Genotype, *n* (%) Allele frequency					
*Hind*III	**H**^−^**H**^−^	**H**^−^**H^+^**	**H^+^H^+^**	**H^+^** **allele**	**H**^−^ **allele**
Control (*n* = 65)	13.0(20%)	23.0(35.4%)	29.0(44.6%)	0.62	0.38
Patient (*n* = 120)	6.0(5%)	53.0(44.2%)	61.0(50.8%)	0.73	0.27
*Pvu*II	**P**^−^**P**^−^	**P**^−^**P^+^**	**P^+^P^+^**	**P^+^** **allele**	**P**^−^ **allele**
Control (*n* = 65)	12.0(18.5%)	28.0(43.0%)	25.0(38.5%)	0.60	0.40
Patient (*n* = 120)	18.0(15%)	52.0(43.3%)	50.0(41.7%)	0.63	0.37
Ser447Ter	**CC**	**CG**		**G allele**	**C allele**
Control (*n* = 65)	57.0(87.7%)	8.0(12.3%)		0.061	0.93
Patient (*n* = 120)	100(83.3%)	20.0(16.7%)		0.083	0.917

*χ* test was used to compare the allele frequencies between control and CAD patients.

**Table 4:** Plasma concentrations of triglycerides (TG), total cholesterol (TC), high density lipoprotein-cholesterol (HDL-c) and low density lipoprotein-cholesterol (LDL-c) in various genotypes of the *Hind*III, *Pvu*II and Ser447Ter in CAD patient group.

Genotype	TG (mmol/L)	TC (mmol/L)	HDL-c (mmol/L)	LDL-c (mmol/L)
*Hind*III				
*H*^−^*H*^−^	1.47 ± 0.28	4.48 ± 1.51	0.96 ± 0.18	3.02 ± 1.23
*H*^−^*H^+^*	1.59 ± 0.71	4.26 ± 0.95	1.01 ± 0.27	2.49 ± 0.82
*H^+^H^+^*	1.53 ± 0.72	4.19 ± 1.18	1.23 ± 1.22	2.51 ± 0.96
*p*-value	0.722	0.664	0.434	0.453
*Pvu*II				
*P*^−^*P*^−^	1.37 ± 0.41	4.57 ± 1.07	1.14 ± 0.37	2.85 ± 0.94
*P*^−^*P^+^*	1.53 ± 0.70	4.09 ± 1.05	1.16 ± 1.23	2.51 ± 0.83
*P^+^P^+^*	1.64 ± 0.75	4.28 ± 1.13	1.05 ± 0.47	2.46 ± 0.98
*p*-value	0.108	0.848	0.453	0.375
Ser447Ter				
*CC*	1.57 ± 0.70	4.28 ± 1.10	1.13 ± 0.96	2.55 ± 0.92
*GC*	1.48 ± 0.66	4.06 ± 1.03	1.03 ± 0.23	2.45 ± 0.93
*p*-value	0.171	0.721	0.708	0.482

**Table 5:** Odds Ratios for CAD Associated With Carriage of *Hin*dIII-, *Pvu*II- and Ser-447/Ter Carrying Genotypes.

	Odds Ratio	95%CI	*p*
*Hin*dIII genotypes			
*Hin*dIII (H^+^H^−^) *vs. Hin*dIII (H^−^H^−^)	4.5	(1.69–14.76)	0.004 *
*Hin*dIII (H^+^H^+^) *vs. Hin*dIII (H^−^H^−^)	4.6	(1.57–13.20)	0.005 *
*Hin*dIII (H^+^H*^+^*) and *Hin*dIII (H^+^H^−^) *vs. Hin*dIII (H^−^H^−^)	4.8	(1.71–13.19)	0.003 *

*Pvu*II genotypes			
*Pvu*II (P^+^P^−^) *vs. Pvu*II (P^−^P^−^)	1.26	(0.53–2.99)	0.60
*Pvu*II (P^+^P^+^) *vs. Pvu*II (P^−^P^−^)	1.33	(0.56–3.20)	0.52
*Pvu*II (P^+^P^+^) and *Pvu*II (P^+^P^−^) *vs. Pvu*II (P^−^P^−^)	1.42	(0.64–3.16)	0.39

Ser-447/Ter genotypes			
Ser-447/Ter CG+GG vs. Ser-447/Ter CC	1.43	(0.59–3.44)	0.43

* *p* < 0.05;

CI = confidence interval.

**Table 6:** Haplotypes analysis of lipoprotein lipase polymorphism in CAD patients and controls.

*Hin*dIII	*Pvu*II	Ser447Ter	Number in CAD patient/120	Number in control/65	*p*
−	−	C	43 (35.83%)	22 (33.85%)	0.787
+	−	C	70 (58.33%)	33 (50.76%)	0.323
−	−	G	12 (10.0%)	1(1.54%)	0.06
−	+	C	45 (37.5%)	27 (41.54%)	0.59
−	+	G	9 (7.5%)	5 (7.69%)	0.96
+	−	G	10 (8.33%)	3 (4.61%)	0.35
+	+	C	94 (78.33%)	42 (64.62%)	0.04
+	+	G	14(11.67%)	6 (9.23%)	0.61

(−) wild-type; (+) mutant in *Hin*dIII and *Pvu*II. (C) wild-type; (G) mutant in Ser-447/Ter. (−/−/C reference haplotype).
